# Effects of whole body vibration on bone properties in growing rats

**DOI:** 10.1080/23335432.2022.2142666

**Published:** 2022-11-16

**Authors:** Akira Minematsu, Yasue Nishii

**Affiliations:** Department of Physical Therapy, Faculty of Health Science, Kio University, 4-2-2 Umaminaka, Koryo-cho, Kitakatsuragi-gun, 635-0832, Japan

**Keywords:** Bone mechanical strength, bone microstructure, growing rats, whole body vibration

## Abstract

This study aimed to examine the continuous effects of whole body vibration (WBV) on bone properties, in growing rats. Fifty 5-week-old male rats were divided into control and experimental groups. Each experimental group underwent WBV at 50 Hz (0.5* g*, 15 min/day, 5 days/week) for 5 or 10 weeks. Bone size, muscle weight and bone mechanical strength of the right tibia were measured. Trabecular bone microstructure, cortical bone geometry and bone mass of the left tibia were analyzed by micro-CT. Serum levels of bone formation/resorption markers were also measured. In rats that underwent 5-week WBV, tibial cortical bone mineral content and cortical bone area significantly increased (p < 0.05), and tibial cortical bone volume, thickness, maximum load, break point and stiffness tended to be increased (p = 0.05–0.09), compared with control rats. In rats that underwent 10-week WBV, stiffness tended to be increased (p = 0.07), and the serum level of osteocalcin decreased, compared with control rats. These findings suggest that 5-week WBV had beneficial effects on bone properties, and that increased bone mineral content and cortical bone geometry may lead to higher bone mechanical strength. Further studies will be needed to determine the optimal conditions of WBV for improving bone properties in the growth stage.

## Introduction

Risk factors for osteoporosis include those which are fixed and modifiable (IOF 2021). Modifiable risk factors can be improved, as most are lifestyle-related, such as exercise, nutrition and the intake of alcohol, coffee and tobacco. The goal of osteoporosis preventive measures is to gain higher peak bone mass during the growth period, because peak bone mass is a significant predictor of future osteoporosis and fracture risk (Specker et al. 2010). An optimal lifestyle that influences peak bone mass and strength is important for decreasing risk of osteoporosis later in life (Weaver et al. 2016). Regular exercise is highly effective for improving bone mass in children and adolescents (Weaver et al. 2016; Xu et al. 2016; Santos et al. 2017; Lombardi et al. 2019), and impact, resistance and weight-bearing exercises are recommended given their beneficial effects on bone health in this population (Kohrt et al. 2004). Recently, whole body vibration (WBV) was reported to improve bone mineral density (BMD) in children, adolescents and postmenopausal women (Slatkovska et al. 2010). Effects of WBV on bone mass may differ by WBV condition, as WBV is considered to improve bone mass depending on the degree of bone strain caused by mechanical loading and muscle contraction. A previous study in postmenopausal women reported that WBV of 3 *g* or higher at a frequency lower than 25 was effective in improving BMD (Fratini et al. 2016). Meanwhile, an increase in bone mass, rather than a decrease in bone loss has been shown to have positive effects of WBV in children and adolescents (Slatkovska et al. 2010). Studies in children and adolescents with disabilities showed that WBV at a frequency of 12–90 Hz or a magnitude of 0.3–12 *g* (Slatkovska et al. 2010; Marin-Puyalto et al. 2018; Swolin-Eide et al. 2020) had positive effects on bone mass. In addition, WBV was found to be more effective on increasing BMD and bone mineral content (BMC) in children and adolescents with compromised bone mass than in postmenopausal women (Marin-Puyalto et al. 2018). However, only a few reports have been published on the effects of WBV on bone mass in normal children/adolescents. One study reported that high magnitude vibration on the arms increased trabecular BMD in the radius (Binkley et al. 2014), but another study found no effects of WBV on BMD or BMC (Gómez-Bruton et al. 2017).

A number of animal studies have examined the effects of WBV on bone properties in normal young rodents (Xie et al. 2006, 2008; Gnyubkin et al. 2016; Zhang et al. 2018). These studies have reported that WBV improves bone mass and structural parameters in young rodents with spinal cord injury, unloading, ovariectomy or osteogenesis imperfecta (Li et al. 2012; Vanleene and Shefelbine 2013; Chen et al. 2014; Minematsu et al. 2016). For instance, in young normal male mice, WBV for 3 weeks increased femoral cortical thickness and cross-sectional area, and when applied for 9 weeks, WBV increased femoral cortical tissue mineral density (TMD) (Gnyubkin et al. 2016). WBV also decreased osteoclastic activity in tibial trabecular bone and increased the bone formation rate of the tibial metaphysis when applied for 3 weeks in young normal female mice (Xie et al. 2006). Moreover, WBV for 5 weeks increased femoral mechanical strength in growing female mice (Vanleene and Shefelbine 2013). As mentioned above, most studies reporting the positive effects of WBV on bone properties in young rodents used frequencies in the range of 30–90 Hz with 0.3 *g* acceleration for 15–20 min/day, 5 days/week (Xie et al. 2006, 2008; Vanleene and Shefelbine 2013; Gnyubkin et al. 2016). Cumulative doses of WBV stimulation ranged from 75 to 100 min/week with a duration of over 225 minutes during the intervention period. In contrast, WBV had no effects on femoral bone mass, structure or mechanical strength when applied for 12 weeks in growing normal female rats (Zhang et al. 2018). Thus, there are inconsistencies in the field regarding the impact of WBV and its effectiveness as a preventive measure against osteoporosis. Sensitivity of bone to WBV likely differs depending on the particular conditions used, including differences in parameters such as frequency, acceleration, duration, term and timing of intervention, as well as the age of rodents, and it is unclear whether WBV has beneficial effects on bone properties of growing rats. A number of animal studies have examined the effects of WBV on bone using low-magnitude, high frequency vibration stimuli, especially in non-invasive growing rodents (Xie et al. 2006; Gnyubkin et al. 2016; Zhang et al. 2018). Mechanical stimuli under these conditions were effective in eliciting a bone response (Rubin et al. 2001). In addition, our previous study (Minematsu et al. 2019) showed that WBV at 45–60 Hz frequency with 0.5 *g* acceleration had a potential to improve bone properties in growing rats. Therefore, the present study used vibration stimuli at 50 Hz and 0.5 *g*. Rats are widely used in bone studies. We used growing rats aged 5 weeks because rat bone grows rapidly for 10 weeks after birth and slowly thereafter, and BMD similarly increases rapidly after birth (Horton et al. 2008). In addition, few studies have examined WBV and bone properties using growing rats (i.e. younger than 5-week-old). Growing bone during and after the rapid growth phase is sensitive and responsive to WBV. This study aimed to examine the effects of WBV for 5 and 10 weeks on tibial bone properties in growing rats aged 5 weeks.

## Materials and Methods

### Animal care and experimental protocol

Fifty 4-week-old male Sprague Dawley rats were purchased from Japan SLC, Inc. (Hamamatsu, Japan). After 1 week of acclimatization, rats were divided into 2 control (CON) and 3 intervention (WBV) groups that were matched according to body weight (BW) and assigned to either 5-week (S: short term) or 10-week (L: long term) experiment period, as follows: S-CON, S-WBV, L-CON, L-WBV and WBV-CON groups (n = 10 each). Rats of the intervention groups underwent WBV for 5 weeks (S-WBV) or 10 weeks (L-WBV) using a vibration device system (Big Wave G-MasterPRO; Asahi Seisakusho Co. Ltd., Tokyo, Japan). This vibration device is a vertical planer type device and can control frequency, acceleration and duration via a personal computer. During WBV, rats were individually placed in a box (L:25 × W:10 × H:10 cm) made of tinted acrylic board over the device. The WBV-CON group had a non-WBV period for 5 weeks after undergoing 5 weeks of WBV. With regard to the control groups, the S-CON group had a 5-week non-WBV period and the L-CON group had a 10-week non-WBV period. WBV condition were as follows: vertical direction vibration, frequency of 50 Hz and acceleration of 0.5 *g* for 15 min/day and 5 days/week. Rats were housed in standard cages in an animal facility where the room temperature and lighting were controlled (temperature, 22–24 °C; lighting, 12:12-h light-dark cycle), and were fed standard rodent chow (CE-2; CLEA Japan Inc., Tokyo, Japan) and water *ad libitum* throughout the experimental period. The study was performed according to the Guide for Animal Experimentation at the Kio University. The animal experimental protocol was approved by the Committee of Research Facilities and Laboratory Animal Science of the Kio University.

Blood, muscles and tibias were collected from all rats at the end of the intervention period (i.e. at 5 and 10 weeks). Serum samples, obtained from blood centrifugation at 1,000 *g* for 25 minutes, were stored at −80°C until biochemical analyses and enzyme-linked immunosorbent assays (ELISA). Bilateral soleus and extensor digitorum longus (EDL) muscles were weighed. After harvesting bilateral tibias, wet weight and length of each tibia were measured. Right and left tibias were stored in saline and 70% ethanol, respectively, until analyzed.

### Analyses of bone mass, trabecular bone microstructure (TBMS) and cortical bone geometry (CBG)

The left proximal metaphysis and diaphysis of the tibia were scanned at 60 kV, 60 µA and a voxel size of 9.6–9.7 µm for TBMS and CBG analysis using an x-ray micro-computed tomography system (Micro-CT; Yamato Scientific Co. Ltd., Tokyo, Japan). The region of interest (ROI) for TBMS of proximal tibia was a 2 mm-length portion of the tibia metaphysis, and the first slice was scanned 0.5 mm distal from the physeal-metaphyseal demarcation. The ROI for CBG was a 2 mm-length portion 2 mm apart from tibiofibular connection. Scanned data were transmitted to a personal computer, and TBMS and CBG of the ROI were analyzed using bone analysis software (TRI BON 3D; Ratoc System Engineering Co. Ltd., Tokyo, Japan). Bone volume fraction (bone volume (BV)/tissue volume (TV)), trabecular bone thickness (Tb.Th), trabecular bone number (Tb.N), trabecular bone separation (Tb.Sp), connectivity density (Conn.D) and trabecular bone pattern factor (TBPf) were assessed as TBMS parameters. Cortical bone volume (Ct.V), medullary volume (MV), cortical bone volume fraction (Ct.V/all bone volume (AV)), cortical bone thickness (Ct.Th), cortical bone sectional area (Ct.Ar), periosteal perimeter (Ps.Pm) and endocortical perimeter (Ec.Pm) were assessed as CBG parameters. A BMD phantom was simultaneously scanned under the same scanning conditions to obtain tissue mineral density (TMD), bone mineral content (BMC) and volume BMD (vBMD; BMC/TV) of the tibial trabecular and cortical bones.

### Measurements of bone mechanical strength

Maximum load and break point of the right tibia were measured by a 3-point bending strength test using a Universal Testing Machine (Autograph AGS; Shimadzu Corp., Kyoto, Japan). Stiffness was also calculated. Bones were supported by 2 fulcrums (5 mm in diameter), and the distance between both fulcrums was half of the bone length. Downward pressure was applied to the center of the bone at a fixed speed of 1 mm/min.

### Dry bone and ash weight measurements

After the tibia was used to measure TBMS parameters, bones were dehydrated in ethanol for 48 hours and then heated at 100°C for 24 hours in a drying machine (Yamato Kagaku, Tokyo, Japan) to obtain dry bone weight. Bones were burned to ash at 600°C for 24 hours with an electric furnace (Nitto Kagaku Co. Ltd., Nagoya, Japan), and the ash content was weighed. Dry bone weight-corrected ash weight was calculated as %ash.

### Serum biochemical analyses and ELISAs

Serum samples were analyzed for calcium, inorganic phosphorus (IP) and alkaline phosphatase (ALP). Serum osteocalcin (OC) and tartrate-resistant acid phosphatase-5b (TRACP-5b) levels were determined with commercially available ELISA kits for OC (Immunodiagnostic Systems Ltd., Boldon, UK) and TRACP-5b (Immunodiagnostic Systems Ltd., Boldon, UK).

### Statistical analysis

All values are expressed as mean ± standard deviation. Differences in the effects of WBV on measured parameters between the S-CON and S-WBV groups were examined by the non-parametric Mann-Whitney U test. Overall differences among long term WBV groups (L-CON, L-WBV and WBV-CON groups) were determined by the non-parametric Kruskal-Wallis test, and differences between individual groups were examined using the Steel-Dwass test. All statistical analyses were performed using Excel Statistics software (BellCurve for Excel version 4.01 for Windows; Social Survey Research Information Co., Ltd., Tokyo, Japan). P < 0.05 was considered statistically significant.

## Results

### Effects of WBV for 5 weeks on bone parameters

**Figure 1.**
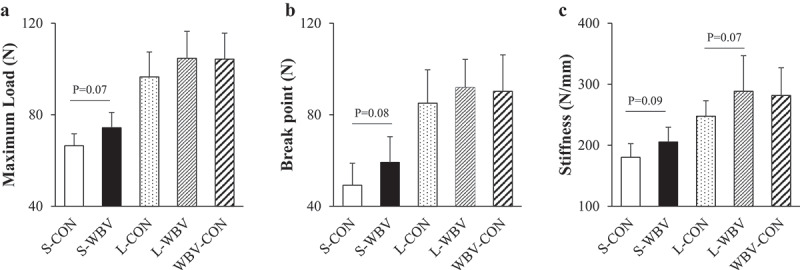
Maximum load (a), break point (b) and stiffness (c) of tibia in all groups. Bar = S.D.

Body weight, daily food intake, wet bone weight and %ash were significantly higher in the S-WBV group than in the S-CON group (Table 1). Cortical BMC was significantly higher in the S-WBV group compared with the S-CON group (Table 2). TBMS parameters did not differ among the 5-week WBV groups (Table 3). As for CBG parameters, Ct.Ar was significantly higher in the S-WBV group compared with the S-CON group, and Ct.V and Ct.Th tended to be higher in the S-WBV group compared with the S-CON group (p = 0.05 and 0.08, respectively) (Table 3). Maximum load, break point and stiffness tended to be higher in the S-WBV group compared with the S-CON group (p = 0.07, 0.08 and 0.09, respectively) (Figure 1). IP was significantly higher in the S-WBV group compared with the S-CON group (Table 4).

**Table 1.** Body weight, food intake, muscle weight and tibial bone size.

	S-CON	S-WBV	L-CON	L-WBV	WBV-CON
Body weight (g)	371.4 ± 14.0	399.3 ± 32.4^a^	493.7 ± 27.8	496.2 ± 22.8	521.2 ± 44.0
Food intake (g/day)	22.9 ± 1.8	24.7 ± 2.6^a^	24.3 ± 2.1	24.7 ± 1.8	25.4 ± 2.7
Muscle weight (mg)					
Soleus	143.2 ± 8.7	148.0 ± 12.0	177.6 ± 16.9	179.2 ± 14.7	188.1 ± 19.0
EDL	170.0 ± 12.3	169.7 ± 14.6	205.0 ± 13.3	220.1 ± 18.6^b^	219.9 ± 20.5^b^
Bone length (mm)	40.0 ± 0.5	40.3 ± 0.8	43.4 ± 1.0	43.5 ± 0.7	43.7 ± 1.0
Wet bone weight (mg)	779.1 ± 44.2	817.0 ± 50.3^a^	940.8 ± 59.3	960.7 ± 60.0	980.9 ± 67.8
Dry bone weight (mg)	471.5 ± 28.7	492.1 ± 30.3	622.2 ± 36.3	636.2 ± 43.9	655.0 ± 47.0
%ash	60.0 ± 0.8	60.9 ± 0.5^a^	62.7 ± 0.7	63.9 ± 0.8^b^	63.2 ± 0.7

Notes: All values are expressed as mean ± SD. EDL, extensor digitorum longus; %ash, ash weight-dry bone weight ratio. ^a^ Significantly different from the S-CON group (p < 0.05). ^b^ Significantly different from the L-CON group (p < 0.05).

**Table 2.** Bone mass parameters of trabecular and cortical bone of tibias.

	S-CON	S-WBV	L-CON	L-WBV	WBV-CON
Trabecular bone					
TMD (mg/cm^3^)	809.8 ± 21.1	815.0 ± 18.1	864.5 ± 25.6	869.7 ± 44.0	895.0 ± 15.2^b^
BMC (mg)	2.36 ± 0.68	2.64 ± 0.78	2.76 ± 0.62	3.04 ± 0.66	3.10 ± 0.75
vBMD (mg/cm^3^)	100.9 ± 24.3	112.3 ± 35.7	139.2 ± 23.2	149.2 ± 19.1	150.5 ± 35.1
Cortical bone					
TMD (mg/cm^3^)	1316.6 ± 15.0	1322.6 ± 15.0	1366.0 ± 11.0	1372.5 ± 9.6	1373.1 ± 15.1
BMC (mg)	10.2 ± 0.8	11.0 ± 1.0^a^	13.2 ± 0.9	13.9 ± 1.3	14.0 ± 0.8

All values are expressed as mean ± SD. TMD, tissue mineral density; BMC, bone mineral content; vBMD, volume bone mineral density. ^a^ Significantly different from the S-CON group (p < 0.05). ^b^ Significantly different from the L-CON group (p < 0.05).

**Table 3.** Trabecular bone microstructure and cortical bone geometry in tibias.

	S-CON	S-WBV	L-CON	L-WBV	WBV-CON
TBMS parameters					
BV/TV (%)	12.4 ± 2.8	13.7 ± 4.2	16.2 ± 2.6	17.2 ± 2.0	16.9 ± 3.8
Tb.Th (µm)	67.5 ± 3.4	68.3 ± 3.1	84.5 ± 3.2	85.2 ± 3.7	84.9 ± 4.9
Tb.N (mm^−1^)	1.56 ± 0.23	1.67 ± 0.39	1.55 ± 0.29	1.66 ± 0.16	1.54 ± 0.32
Tb.Sp (µm)	209.1 ± 16.3	208.5 ± 25.8	193.3 ± 17.7	190.9 ± 9.9	194.0 ± 16.1
Conn.D (mm^−3^)	28.7 ± 11.5	35.5 ± 19.77	34.3 ± 10.1	37.2 ± 7.2	38.8 ± 13.1
TBPf (mm)	18.0 ± 2.7	16.2 ± 3.2	13.7 ± 1.4	13.3 ± 1.1	13.0 ± 2.1
CBG parameters					
Ct.V (mm^3^)	7.75 ± 0.56	8.32 ± 0.71	9.63 ± 0.74	10.14 ± 0.93	10.22 ± 0.66
MV (mm^3^)	2.33 ± 0.29	2.36 ± 0.24	2.46 ± 0.23	2.30 ± 0.33	2.38 ± 0.32
Ct.V/AV (%)	76.9 ± 2.2	77.9 ± 1.2	79.6 ± 1.9	81.5 ± 2.4	81.1 ± 2.1
Ct.Th (µm)	657.3 ± 36.7	689.5 ± 32.0	756.9 ± 45.0	800.8 ± 58.4	802.5 ± 42.8
Ct.Ar (mm^2^)	3.88 ± 0.27	4.16 ± 0.35^a^	4.82 ± 0.37	5.07 ± 0.47	5.11 ± 0.34
Ps.Pm (mm)	8.29 ± 0.29	8.48 ± 0.36	9.12 ± 0.36	9.25 ± 0.35	9.30 ± 0.36
Ec.Pm (mm)	3.99 ± 0.23	4.01 ± 0.19	4.20 ± 0.26	4.06 ± 0.35	4.07 ± 0.31

All values are expressed as mean ± SD. BV/TV, trabecular bone volume fraction; Tb.Th, trabecular bone thickness; Tb.N, trabecular bone number; Tb.Sp, trabecular bone separation; Conn.D, connectivity density; TBPf, trabecular bone pattern factor; Ct.V, cortical bone volume; MV, medullary volume; Ct.V/AV, cortical bone volume fraction; Ct.Th, cortical bone thickness; Ct.Ar, cortical bone sectional area; Ps.Pm, periosteal perimeter; Ec.Pm, endocortical perimeter. ^a^ Significantly different from the S-CON group (p < 0.05).

**Table 4.** Serum biochemical analysis.

	S-CON	S-WBV	L-CON	L-WBV	WBV-CON
Ca (mg/dL)	10.3 ± 0.2	10.4 ± 0.2	10.5 ± 0.3	10.4 ± 0.2	10.6 ± 0.3
IP (mg/dL)	7.3 ± 0.2	7.9 ± 0.5^a^	6.0 ± 0.2	6.1 ± 0.4	6.4 ± 0.3^b^
ALP (mg/dL)	1138.8 ± 219.5	1295.5 ± 331.0	874.6 ± 127.5	914.8 ± 231.0	768.2 ± 121.7
Osteocalcin (ng/mL)	833.8 ± 78.7	804.1 ± 82.6	459.5 ± 59.7	385.6 ± 48.8^b^	337.0 ± 64.0^b^
TRACP-5b (U/L)	8.8 ± 1.7	9.1 ± 3.4	6.9 ± 1.1	7.2 ± 1.1	6.1 ± 0.6^c^

All values are expressed as mean ± SD. Ca, calcium; IP, inorganic phosphorus; ALP, alkaline phosphatase; TRACP-5b, tartrate-resistant acid phosphatase-5b. ^a^ Significantly different from the S-CON group (p < 0.05). ^b^ Significantly different from the L-CON group (p < 0.05). ^c^ Significantly different from the L-WBV group (p < 0.05).

*Siginicantly different from the S-CON group (p < 0.05).

### Effects of WBV for 10 weeks on bone parameters

There was no difference in body weight and daily food intake among the 10-week experimental groups (Table 1). EDL muscle weight was significantly larger in the L-WBV and WBV-CON groups than in the L-CON group, and %ash was significantly higher in the L-WBV group than in the L-CON group. TMD of trabecular bone was significantly higher in the WBV-CON group than in the L-CON group (Table 2). TBMS and CBG parameters did not differ among the 10-week WBV groups (Table 3). Maximum load and break point were higher in the L-WBV group compared with the L-CON group, although the difference was not significant (Figure 1). Stiffness tended to be higher in the L-WBV group (p = 0.07) compared with the L-CON group (Figure 1). IP was significantly higher in the WBV-CON group compared with the L-CON group (Table 4). The serum level of osteocalcin was lower in the L-WBV and WBV-CON groups compared with the L-CON group (Table 4). The serum level of TRACP-5b was significantly lower in the WBV-CON group compared with the L-WBV group (Table 4).

## Discussion

In the present study, WBV at a frequency of 50 Hz with an acceleration of 0.5 *g* for 5 weeks significantly increased cortical BMC and Ct.Ar in growing male rats. WBV had positive effects on CBG parameters (e.g. Ct.V and Ct.Th) and bone mechanical strength (maximum load, break point and stiffness). WBV was especially effective in improving cortical bone properties, and these changes likely contributed to an increase in bone mechanical strength.

In growing rodents, WBV has been reported to have beneficial effects on bone, including increased femoral trabecular bone cellular activity, femoral Ct.Th and Cr.Ar (90 Hz, 2 *g*, for 3 weeks) (Gnyubkin et al. 2016); an increase in bone formation in the tibial metaphysis and decrease in osteoclastic activity in tibial trabecular bone (45 Hz, 0.3 *g*, for 3 weeks) (Xie et al. 2006); an increase of bone mineralizing surface in tibial trabecular bone, tibial trabecular BV and tibial Cr.Ar (45 Hz, 0.3 *g*, for 6 weeks) (Xie et al. 2008); and an increase in stiffness and yield force of femur (45 Hz, 0.3 *g*, for 5 weeks) (Vanleene and Shefelbine 2013). Thus, short term WBV has positive effects on bone cells and the structure and mechanical strength of bone. In the present study, WBV for 5 weeks significantly increased cortical BMC and Ct.Ar of the tibia compared with the control group. Short term WBV has been reported to increase Ct.Th and Ct.Ar of the tibia and femur (Xie et al. 2008; Vanleene and Shefelbine 2013; Gnyubkin et al. 2016), and stiffness and yield force of the femur were increased in 3-week-old wild type mice that underwent WBV for 5 weeks (Vanleene and Shefelbine 2013). In addition, WBV (45 Hz, 0.3 *g*) for 6 weeks, but not 3 weeks, was reported to increase Ct.Ar in 8-week-old mice (Xie et al. 2006, 2008). The increase in Ct.Ar observed in the present study is consistent with the previous reports (Xie et al. 2006, 2008; Vanleene and Shefelbine 2013; Gnyubkin et al. 2016). In addition, Ct.V and Ct.Th tended to be increased, which possibly led to the increase in cortical BMC. Therefore, the increases in cortical BMC and CBG (Ct.V, Ct.Th, and Ct.Ar) observed in the present study due to 5 weeks of WBV may have contributed to the increase in mechanical strength of the tibia, although not significantly.

Rat bone grows rapidly for 10 weeks after birth and slowly thereafter, and BMD similarly increases rapidly after birth (Horton et al. 2008). Effects of loading on bone mass and structure are the most beneficial during the rapid growth stage (Mosley and Lanyon 2002). In addition, low magnitude WBV reportedly has beneficial effects on bone properties in osteopenic models (Li et al. 2012; Vanleene and Shefelbine 2013; Chen et al. 2014; Minematsu et al. 2016). Thus, the response of bone to stimulus may be more sensitive when bone cells or bone metabolism changes are active, even when the magnitude of the stimulus is low. In addition, stimulating bone in the growing stage can activate bone cells and increase TMD or bone size. Higher Ct.Th and Ct.Ar may contribute to an increase in bone mechanical strength parameters in growing rats. In this regard, we found that WBV for 5 weeks increased bone mechanical strength, likely due to increases in Ct.V, Ct.Th, Ct.Ar and BMC.

As opposed to WBV for 5 weeks, WBV for 10 weeks did not significantly increase bone mechanical strength compared with the L-CON group in the present study. WBV for 12 weeks (45 Hz, 0.3 *g*) in 4-week-old female rats has been reported to have no effects on bone properties (Zhang et al. 2018). Similarly, other studies have reported that WBV did not impact bone properties in normal young or adult rats (Rubinacci et al. 2008; Lynch et al. 2011), although it had positive effects on bone properties of young osteopenia model rats (Rubinacci et al. 2008; Sehmisch et al. 2009; Tezval et al. 2011; Kakihata et al. 2020). WBV for 9 weeks has also been reported to have no effect on bone properties, with the exception of femoral cortical TMD, although WBV for 3 weeks had positive effects on bone formation rate, mineral apposition rate, Ct.Th and Ct.Ar in 7-week-old mice (Gnyubkin et al. 2016). Results from these previous studies are similar to our present results, although long-term WBV had no effect on cortical TMD in the present study. The discrepancy regarding the effect of long-term WBV on cortical TMD was likely due to differences in WBV conditions, as the previous study applied WBV at 90 Hz with 2 *g* acceleration (Gnyubkin et al. 2016). As discussed above, bone growth in rats slows after age 10 weeks (Horton et al. 2008), and adult bone is considered to be less responsive to WBV. In fact, low magnitude WBV did not affect bone properties in adult rodents (Rubinacci et al. 2008; Lynch et al. 2011; Zhang et al. 2018). In contrast, bone in young mice has been reported to respond to low strain loading, although the impact of high strain loading on increasing bone formation and decreasing bone resorption is observed not only in young mice, but also in adult and old mice (Birkhold et al. 2014; Razi et al. 2015). Moreover, WBV at a lower frequency (less than 10 Hz) has been reported to cause deterioration of bone, whereas in normal mature rat, WBV at higher frequency had positive effects on bone (Pasqualini et al. 2013). In humans, WBV improves BMD in children and adolescents, but not young adults (Slatkovska et al. 2010). These results suggest that young adult bone may have a higher threshold for WBV than growing bone.

The mechanism of bone property improvement by WBV is likely to involve mechanical stress and muscle contraction. In a previous study, the maintenance of bone mass was reported to depend on both the magnitude of daily stress/strain and number of loading cycles, according to the daily stress stimulus theory (Qin et al. 1998). The number of daily cycles was 45,000 in the present study. According to Qin et al., the resultant strain magnitude required to maintain bone mass was calculated to be approximately 175 µɛ, and approximately 30,000 to 100,000 cycles per day would be needed to achieve this strain (Qin et al. 1998). In addition, a higher acceleration was used in the present study (0.5 *g*) compared with the previous study (Qin et al. 1998). Muscle contraction has also been reported to influence bone metabolism (Avin et al. 2015; Tagliaferri et al. 2015), and effective frequency on bone of 20–50 Hz was previously reported (Lam and Qin 2008; Qin et al. 2010). WBV conditions (i.e. 50 Hz frequency with 0.5 *g* acceleration) used in the present study may have been sufficient to affect CBG in the rapid bone growth phase (i.e. for the first 5 weeks), whereas the bone response to WBV may have been less pronounced in the slow bone growth phase. Similar to the results of the present study, positive effects of WBV for less than 5 weeks on bone were observed in normal young rodents (Xie et al. 2006, 2008; Vanleene and Shefelbine 2013; Gnyubkin et al. 2016). WBV stimuli in the rapid bone growth phase may activate bone adaptation processes, which may in turn improve the properties of cortical bone. However, WBV for 10 weeks had no significant effects on bone properties, although average values were higher in the L-WBV group compared the L-CON group. Similarly, Ct.Th and Ct.Ar after WBV for 9 weeks did not differ from those after WBV for 3 weeks in age-matched controls, suggesting that a steady state might have been achieved in the adaptation of the skeleton to this mechanical environment (Gnyubkin et al. 2016). In the present study, no carryover effects of WBV for 10 weeks on bone properties were observed, possibly because the initial WBV stimulus reached a steady state of bone adaptation, and more stimulus was needed to improve bone properties. Our findings suggest that the effects of WBV on bone properties differ by the period of WBV.

In the present study, WBV for 5 weeks did not affect serum TRACP-5b and OC levels, but increased serum ALP levels, albeit not significantly. On the other hand, WBV for 10 weeks decreased serum OC levels and increased serum ALP levels, albeit not significantly, while serum TRACP-5b levels remained unchanged. A previous study reported that WBV has negative effects on bone properties, including decreased serum TRACP-5b and ALP levels (Zhang et al. 2018). In another study, serum OC levels declined, with no change in serum TRACP-5b levels, after 35 days of age (Horton et al. 2008). Thus, the decrease in serum OC levels after 10 weeks of WBV may be due to aging. WBV induces both bone formation and resorption, likely because bone remodeling and modeling are active in growing rodents (Birkhold et al. 2014; Razi et al. 2015). This suggests that WBV may influence bone-related hormones and/or RANKL (Prisby et al. 2008; Wei et al. 2014; Zhou et al. 2015), in addition to bone histological changes such as bone formation rate, mineral apposition rate, and bone blast and clast numbers.

## Limitation

This study has some limitations. First, only one frequency (50 Hz) with one magnitude (0.5 *g*) was used for WBV intervention. Second, we did not measure dynamic strain in the tibia, which could have been estimated from measurements in previous studies (Mosley and Lanyon 2002; Xie et al. 2006; Pasqualini et al. 2013). Finally, bone-related hormones and cytokines were not assessed, and histological measurements were not performed, although bone formation and resorption markers were analyzed, Further studies are needed to better understand the effects of WBV on hormones and cytokine secretion, as well as its influence on bone properties.

## Conclusions

Our findings suggest that WBV at 50 Hz for 5 weeks significantly increased cortical BMC and Ct.Ar of the tibia in normal rats from 5 weeks of age. WBV had positive effects on Ct.V, Ct.Th, and bone mechanical strength (maximum load, break point and stiffness), and these changes could lead to an increase in bone mechanical strength. However, carry-over effects of long term WBV on tibial mechanical strength were not observed. In order to fully benefit from the bone-enhancing properties of WBV, further studies will be needed to determine the optimal timing and duration of WBV, in addition to WBV conditions.

## Data Availability

The data that support the findings of this study are available from the corresponding author, A.M., upon reasonable request. Requests to access the data should be directed to AM, a.minematsu@kio.ac.jp.
